# A Rare Cause of Unexplained Refractory Iron Deficiency Anemia: Unicentric Plasma-Cell Type Castleman’s Disease

**DOI:** 10.4274/tjh.2016.0083

**Published:** 2016-08-19

**Authors:** Sevgi Kalayoğlu Beşışık, İpek Yönal Hindilerden, Fehmi Hindilerden, İbrahim Öner Doğan, Fatih Beşışık

**Affiliations:** 1 İstanbul University İstanbul Faculty of Medicine, Department of Internal Medicine, Division of Hematology, İstanbul, Turkey; 2 İstanbul Bakırköy Sadi Konuk Training and Research Hospital, Clinic of Hematology, İstanbul, Turkey; 3 İstanbul University İstanbul Faculty of Medicine, Department of Pathology, İstanbul, Turkey; 4 İstanbul University İstanbul Faculty of Medicine, Department of Internal Medicine, Division of Gastroenterohepatology, İstanbul, Turkey

**Keywords:** Iron deficiency anemia, Unicentric plasma-cell type, Castleman’s disease

## To the Editor,

Castleman’s disease (CD) is an uncommon benign lymphoproliferative disorder characterized by enlargement of hyperplastic lymph nodes with abnormal interfollicular vascular growth [1]. It is clinically categorized as unicentric disease or multicentric disease. Histopathologic variants of CD include hyaline-vascular type, plasma-cell (PC) type, and mixed form [2]. CD presents with features ranging from asymptomatic lymphadenopathy to systemic manifestations such as serious infections, anemia, and nerve damage [3]. In CD, hepcidin secretion induced by IL-6 is the main cause of anemia [4,5]. Anemia is more common in multicentric CD and is rarely reported in unicentric CD [3]. Few cases of unicentric PC type CD associated with iron deficiency anemia (IDA) have been reported [6,7,8,9]. To our knowledge, there is only one previous reported case of adult unicentric PC type CD located in the abdomen and presenting with IDA [9]. We describe an adult with occult unicentric PC type CD of the abdomen presenting with iron-refractory anemia (IRA) and achieving dramatic response to curative resection.

**Figure 1:**
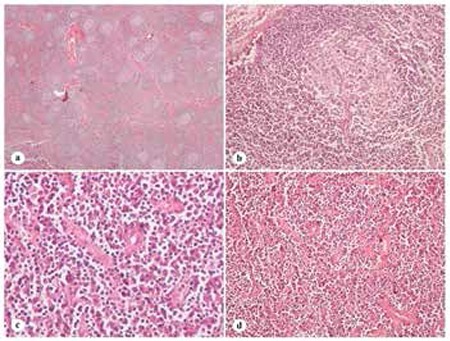
Histopathological examination of the resected specimen. Greater retention of the nodal architecture with increased secondary lymphoid follicles, and vascularization of germinal centers (a: 40^x^), germinal centers fed by prominent vessels; lollipop-like appearance (b: 200^x^). The interfollicular region contained sheets of mature-appearing plasma cells and increased postcapillary venules (c: 400^x^; d: 200^x^).

A 47-year-old man was referred with a 5-year history of IRA. Blood analysis showed Hb of 8 g/dL, mean corpuscular volume of 70 fL, red cell distribution width of 17%, and platelet count of 478,000/mm^3^. Biochemical tests were as follows: serum ferritin, 371 µg/L; transferrin saturation, 8.6%; and erythrocyte sedimentation rate (ESR), 110 mm/h (reference range: 0-20). Serum protein electrophoresis revealed polyclonal gammopathy with gamma globulin of 2.16 g/dL. The soluble transferrin receptor/log10 ferritin index of 2.3 indicated the presence of combined IDA and anemia of inflammation (AI) [10]. Underlying chronic inflammatory diseases were excluded. Upper and lower gastrointestinal endoscopic evaluations and bone marrow examination were normal. Positron emission tomography-computed tomography showed a soft tissue mass with diffuse fluorodeoxyglucose uptake with an SUVmax of 11.39 and a craniocaudal length of 8 cm extending from the hepatogastric ligament and with a maximal diameter of 4.4x4.3 cm in the axial plane at the portal region. He underwent exploratory laparotomy and the mass was completely excised. Histopathological examination of the mass revealed greater retention of the nodal architecture with increased secondary lymphoid follicles, vascularization of germinal centers, and expansion of mantle zones. The interfollicular region contained sheets of CD38-positive mature-appearing plasma cells and increased postcapillary venules. Plasma cells expressed polytypic immunoglobulins, light and heavy chains (Figure 1). These findings were compatible with a diagnosis of PC type CD. Four months after complete resection, laboratory tests had completely normalized (Hb 14 g/dL, mean corpuscular volume 83 fL, and ESR 10 mm/h). He has been free of disease for more than 26 months since the resection.

Previously reported PC type CD patients with abdominal involvement and IDA aged between 11 and 29 years [6,7,9], making our patient the oldest reported patient. PC type CD with abdominal involvement may show an indolent course, often leading to great delay in diagnosis. We report an unusual case of unicentric PC type CD in which the patient suffered from systemic manifestations of anemia for five years. After surgical resection, the anemia completely resolved. CD should be included in the differential diagnosis of chronic, unexplained inflammation associated with combined IDA and AI.

## Ethics

Informed Consent: It was taken.

## References

[1] Castleman B, Iverson L, Menendez VP (1956). Localized mediastinal lymph node hyperplasia resembling thymoma. Cancer.

[2] Keller AR, Hochholzer L, Castleman B (1972). Hyaline-vascular and plasma-cell types of giant lymph node hyperplasia of the mediastinum and other locations. Cancer.

[3] Bjarnason I, Cotes PM, Knowles S, Reid C, Wilkins R, Peters TJ (1984). Giant lymph node hyperplasia (Castleman’s disease) of the mesentery. Observations on the associated anemia. Gastroenterology.

[4] Yoshizaki K, Matsuda T, Nishimoto N, Kuritani T, Taeho L, Aozasa K, Nakahata T, Kawai H, Tagoh H, Komori T (1989). Pathogenic significance of interleukin-6 (IL-6/BSF-2) in Castleman’s disease. Blood.

[5] Song SN, Tomosugi N, Kawabata H, Ishikawa T, Nishikawa T, Yoshizaki K (2010). Down-regulation of hepcidin resulting from long-term treatment with an anti-IL-6 receptor antibody (tocilizumab) improves anemia of inflammation in multicentric Castleman disease. Blood.

[6] Yin L, Lu XY, Xu F, Li AJ, Wu MC (2012). Unicentric Castleman’s disease presenting with growth retardation and iron deficiency anemia. Am J Med Sci.

[7] Chandrakasan S, Bakeer N, Mo JQ, Cost C, Quinn CT (2014). Iron-refractory microcytic anemia as the presenting feature of unicentric Castleman disease in children. J Pediatr.

[8] Suh JH, Hong SH, Jeong SC, Park CB, Choi KB, Shin OR, Choi SY (2015). Anemia resolved by thoracoscopic resection of a mediastinal mass: a case report of unicentric Castleman’s disease. J Thorac Dis.

[9] Vinzio S, Ciarloni L, Schlienger JL, Rohr S, Méchine A, Goichot B (2008). Isolated microcytic anemia disclosing a unicentric Castleman disease: The interleukin-6/hepcidin pathway?. Eur J Intern Med.

[10] Punnonen K, Irjala K, Rajamäki A (1997). Serum transferrin receptor and its ratio to serum ferritin in the diagnosis of iron deficiency. Blood.

